# Ethyl carbamate in Swedish and American smokeless tobacco products and some factors affecting its concentration

**DOI:** 10.1186/s13065-018-0454-x

**Published:** 2018-07-24

**Authors:** K. McAdam, C. Vas, H. Kimpton, A. Faizi, C. Liu, A. Porter, T. Synnerdahl, P. Karlsson, B. Rodu

**Affiliations:** 1 Group Research & Development, British American Tobacco, Regents Park Road, Southampton, SO15 8TL UK; 2 3810 St. Antoine W, Montreal, QC H4C 1B4 Canada; 3 Eurofins Food & Feed Testing Sweden AB, Sjöhagsgatan 3, 531 40 Lidköping, Sweden; 4 Department of Medicine, School of Medicine, University of Louisville, Room 208, 505 South Hancock Street, Louisville, KY 40202 USA

**Keywords:** Ethyl carbamate, Urethane, Smokeless tobacco products, Snus, Snuff

## Abstract

**Background:**

We are interested in comparing the levels of harmful or potentially harmful constituents in Swedish and American smokeless tobacco products (STPs). We report here the concentrations of the IARC Group 2 A (probable human) carcinogen ethyl carbamate (EC) in seventy commercial STPs from the US and Sweden, representing 80–90% of the market share of the major STP categories in these countries. We also examine the effects of various additives, processing and storage conditions on EC concentrations in experimental snus samples.

**Results:**

EC was determined from aqueous extracts of the STPs using ultra performance liquid chromatography tandem mass spectrometry (UPLC/MS/MS). EC was undetectable (< 20 ng/g wet weight basis WWB) in 60% of the commercial STPs, including all the chewing tobacco (CT), dry snuff (DS), hard pellet (HP), soft pellet (SP), and plug products. Measurable levels of EC were found in 11/16 (69%) of the moist snuff (MS) samples (average 154 ng/g in those samples containing EC) and 19/32 (59%) of the Swedish snus samples (average 35 ng/g). For the experimental snus samples, EC was only observed in ethanol treated samples. EC concentrations increased significantly with ethanol concentrations (0–4%) and with storage time (up to 24 weeks) and temperature (8 °C vs 20 °C). EC concentrations were lower at lower pHs but were unaffected by adding nitrogenous precursors identified from food studies (citrulline and urea), increasing water content or by pasteurisation. Added EC was stable in the STP matrix, but evaporative losses were significant when samples were stored for several weeks in open containers at 8 °C.

**Conclusions:**

EC was found in measurable amounts only in some moist STPs i.e. pasteurised Swedish snus and unpasteurised US MS; it is not a ubiquitous contaminant of STPs. The presence of ethanol contributed significantly to the presence of EC in experimental snus samples, more significantly at higher pH levels. Sample age also was a key determinant of EC content. In contrast, pasteurisation and fermentation do not appear to directly influence EC levels. Using published consumption rates and mouth level exposures, on average STP consumers are exposed to lower EC levels from STP use than from food consumption.

**Electronic supplementary material:**

The online version of this article (10.1186/s13065-018-0454-x) contains supplementary material, which is available to authorized users.

## Introduction

Although the International Agency for Research on Cancer (IARC) has categorised STPs collectively as Group 1 (known human) carcinogens [1], there is growing evidence from epidemiologic studies that different types of STPs have different health risks [2]. In the US, the low moisture tobacco powder known as dry snuff (DS), the higher water-content product known as moist snuff (MS) and the various forms of predominately high sugar, low water-content chewing tobacco (CT) are the styles of STP that have been used historically, while products such as American snus and various pellet products have been introduced more recently. In Sweden snus, a high-water content pasteurised tobacco product is the dominant STP. In reviews of the comparative health effects of different styles of STP, users of Swedish snus and American MS and CT products appear to have lower risks of oral cavity cancer than users of American DS products [2, 3]. Knowledge of hazardous or potentially hazardous constituents in STPs is therefore of great scientific and public health interest. For this reason, we have undertaken the analysis of a wide variety of toxicants in STPs used in Scandinavia and North America as previously published [4–7].

In a 2007 monograph, IARC listed 27 carcinogenic or potentially carcinogenic toxicants that had been identified in STPs [1, p. 58–59]. The list included not only the relatively well-studied tobacco specific nitrosamines and polycyclic aromatic hydrocarbons (PAH) but also several toxicants for which there is very limited information, including ethyl carbamate (EC). In 2012 the US Food and Drug Administration (FDA) included EC in its Established List of 93 harmful or potentially harmful constituents (HPHC) of tobacco products, some of which are required to be reported to the FDA [8]. This list covers both tobacco and tobacco smoke components and includes 79 that are designated as carcinogenic, and others that are respiratory toxicants, cardiovascular toxicants, reproductive toxicants or addictive.

EC, or urethane, is the ethyl ester of carbamic acid with the formula NH_2_COOC_2_H_5_. It is a colourless solid with a melting point of 48–50 °C, a boiling point of 182–184 °C [9] and a measurable vapour pressure at room temperature. It is soluble in water and in a wide range of organic solvents. EC has low mutagenicity in bacterial cells and gives positive responses in some mammalian cell assays for chromosomal aberrations, sister chromatid exchange and micronucleus induction [9]. Although there are no relevant epidemiologic studies of human exposure, oral administration of EC to rodents has been shown to induce tumours in various organs, probably via the formation of the metabolite vinyl carbamate and its epoxide [9]. Based on animal studies and mechanistic considerations the IARC has classified EC as a Group 2A (probable human) carcinogen [9].

EC is produced as a naturally occurring by-product of fermentation. It can be found in low concentrations in fermented food products such as bread, soy sauce, yogurt and alcoholic beverages. IARC [9] and the European Food Safety Authority [10] have summarised typical levels of EC in various foodstuffs and alcoholic beverages. For example, the median level in untoasted bread is 2.8 ng/g, which rises to 4.3 and 15.7 ng/g when lightly and darkly toasted. Cheeses contain up to 5 ng/g, while lower levels (< 1 ng/g) are found in yogurts. Soy sauces contain up to 129 ng/g, with higher concentrations found in Japanese-style products. Median (and maximum) concentrations found in alcoholic beverages originating from Europe were 0–5 (33) ng/g for beer (depending on whether undetectable levels were assigned a value of zero or LOD), 5 (180) ng/g for wine, 21 (6000) ng/g for spirits and 260 (22,000) ng/g for stone fruit brandy. Sake samples contained a mean of 98 ng/g of EC with a maximum of 202 ng/g.

**Fig. 1:**
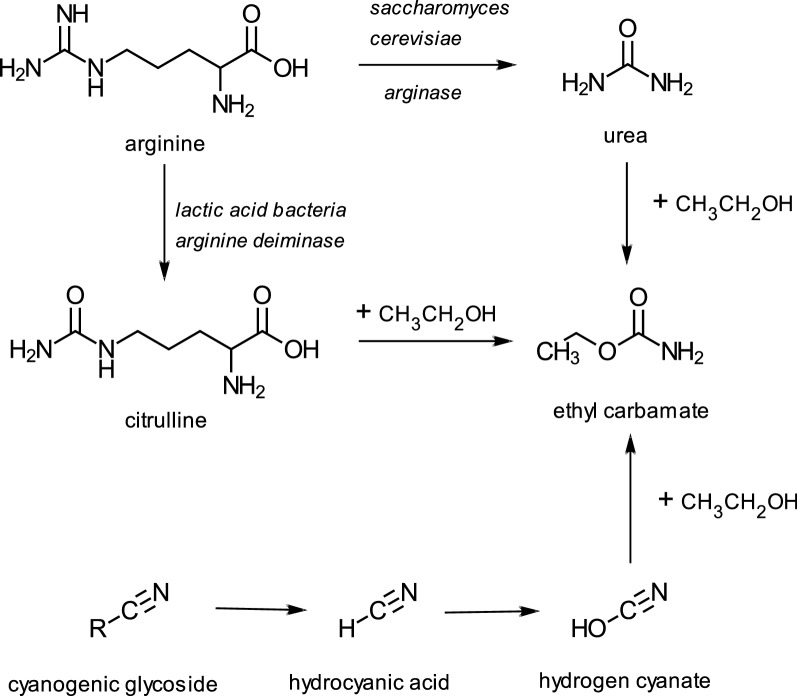
Some pathways to ethyl carbamate in alcoholic beverages after Jiao et al. [48] and [12]

EC is generally thought to be formed in these products by the reaction of various precursors with ethanol (Fig. 1). For alcoholic beverages such as grape wine, rice wine and sake, the major precursor is urea derived from arginine during yeast fermentation [11]. For stone fruit brandies, in particular, an additional precursor is cyanide, derived from cyanogenic glycosides such as amygdalin. Citrulline, derived from the catabolism of arginine by lactic acid bacteria, is also a precursor for EC in wines [12] as well as in soy sauce, in which ethanol present in the fermented soy reacts with citrulline during the pasteurisation process to form EC [13].

In 1986, Canada was the first country to introduce limits on the concentrations of EC in alcoholic beverages [10]. Upper limits for EC were 30 ng/g for wine, 100 ng/g for fortified wine, 150 ng/g for distilled spirits, 200 ng/g for sake and 400 ng/g for fruit brandy. Since then the US and some European Union member states have introduced maximum levels, but there are currently no harmonised maximum EC levels in the European Union.

EC was first reported in two samples of burley tobacco by Schmeltz et al. in 1978 [14]. One, which had been treated with maleic hydrazide, contained 310 ng/g while the other sample, which was untreated, contained 375 ng/g, with both concentrations on a wet weight basis (WWB). These results were subsequently, and erroneously, reported as being obtained from CT [15] or from fermented Burley tobacco [1, p. 60]. Since then there have been several published and unpublished studies of EC in tobacco samples. Clapp [16] and Clapp et al. [17] reported that EC concentrations in the tobacco blends of two US brands of cigarettes were below 10 ng/g (WWB), which was the limit of quantification (LOQ). In an unpublished report, Schroth [18] measured concentrations of EC in 13 German cigarette tobacco blends, ten of which had concentrations below the limit of detection (LOD, 0.7 ng/g WWB) and the other three with concentrations of between 1.4 and 2.9 ng/g WWB. Teillet et al. [19] found no EC in 23 commercial cigarette blends and in seven commercial fine-cut smoking tobacco blends, and Lachenmeier et al. [20] could not detect EC in a tobacco liqueur derived from tobacco leaves. Oldham et al. [21] failed to detect EC in 15 brands of US MS, using a method with an LOD of 90 ng/g (WWB). In another recent study, Stepan et al. [22] measured EC concentrations in a number of tobacco samples using ultra performance liquid chromatography tandem mass spectrometry (HPLC-APCI-MS/MS). The samples consisted of four reference STPs (CRP1—a Swedish style portion snus, CRP2—a US MS, CRP3—a US DS and CRP4—a US CT), 30 commercial STPs and two reference cigarette tobaccos. The LOQ and LOD varied between samples according to moisture content, but when expressed on a dry weight basis (DWB) were found to be reasonably consistent at 200 and 60 ng/g, respectively. Of the reference STPs, only CRP2 (MS) had a detectable concentration of EC (38 ng/g WWB); neither of the reference cigarette tobaccos showed measurable levels of EC. Of the 30 commercial STPs, 17 had no detectable EC, 12 contained EC below the LOQ, and 1 STP had an EC content of 162 ng/g WWB.

Given the lack of understanding of EC in tobacco, a two-part study of EC in STPs was undertaken. The first part was a survey of EC concentrations in 70 STPs from Sweden and the US. These products included loose (L) and portion (P) snus products from Sweden, and CT, DS, MS, hard pellet (HP), soft pellet (SP) and plug products from the US. Based on the results and tentative conclusions of this survey we designed and conducted a series of tests on experimental snus samples to determine the effects of processing variables, additives and storage conditions on EC concentrations.

## Experimental

### Brands of STP included in the survey

STP samples for the survey were obtained in 2009. Products were chosen to reflect a significant proportion of the market segment for each STP category (Additional file 1, Tables S1a and S1b). US market share data were obtained from a commercially available report [23], and Swedish product market shares were acquired using market monitoring by British American Tobacco (BAT) staff. In total, the survey comprised 32 Swedish products (10 L snus and 22 P snus) and 38 US products (13 CT, 5 DS, 2 HP, 1 SP, 16 MS, and 1 plug product). The Swedish products were sourced from Swedish retail websites, transported under ambient conditions, imported into the United Kingdom, and frozen at − 20 °C until analysis. The US products were sourced from shops in the United States, transported under ambient conditions, imported, and frozen at − 20 °C until analysis. Product age at time of sampling is unknown. Clearly, a one-point-in-time sampling regime of this kind does not provide insight into the long-term chemistry of any individual STP. However, by sampling the major products for each category we were able to discuss the EC contents of the product category as a group at the time of sampling. Products sampled represented approximately 88% of the Swedish snus market, 94% of the American CT market, 96% of the American MS market and 51% of the American DS market. The single plug product analysed has a 33% market share. Market shares of the pellet products were not available.

### Snus samples used in controlled laboratory experiments

Four different snus variants (A, B, C and D) were manufactured by Fiedler and Lundgren, Sweden, with different compositions and/or processing conditions in order to examine the following experimental variables.

1. Storage time post-manufacture: up to 24 weeks.
2. Storage temperature post-manufacture: 8 ± 1 and 20 ± 2 °C.
3. Ethanol addition: 0–4%.
4. Urea addition: 0 and 1%.
5. Citrulline addition: 0 and 1%.
6. pH: 8.5 (normal) and 5.5 (treated with citric acid); with and without sodium carbonate.
7. Evaporation during storage: closed vs open container.

Snus A consisted of unpasteurised tobacco, with no sodium carbonate and with approximately 33% water. Snus B contained pasteurised tobacco, with no sodium carbonate and with approximately 44% water. Snus samples C and D were derived from the same pasteurised snus sample containing sodium carbonate. The only difference between C and D was that C contained about 55% water, while snus D was dried to about 15% water.

Subsamples were treated after manufacture with ethanol, EC, urea, citrulline or citric acid (or combinations of these). Urea, citric acid and EC were added in aqueous solution. Citrulline, which is insoluble in water at neutral pH, was added as a powder. Each sample in these studies was analysed for EC in triplicate, with each replicate consisting of 50 g of the snus.

## Methods

We describe below analytical methodology used to generate the data in this study. EC was the main focus of the study, and the method described below was used in both market survey and controlled laboratory studies. The concentrations of a number of other STP constituents were also measured for the market survey samples in an attempt to understand product parameters that influence EC content. These parameters were water content by Karl Fisher, water activity, nicotine, total nicotine alkaloids, total sugars, propylene glycol, glycerol, nitrate, sodium and chloride ions; methodology used to measure these parameters is also described below. Finally, concentrations of reducing sugars, ammonia nitrogen and pH reported previously from the same market survey [6] were also used to identify factors potentially related to EC formation; methods for these parameters were described earlier [6].

### Ethyl carbamate

Eurofins Sweden Ltd. extracted and analysed the STPs using ultra performance liquid chromatography tandem mass spectrometry (UPLC/MS/MS). The aqueous extracts were prepared by placing 4 g samples of the STP in 50 ml polypropylene tubes to which 100 µl of internal standard (EC-D5, 10 µg/ml) and 20 ml of MilliQ filtered water were added. The mixture was shaken for 30 min and then centrifuged at 4000 rpm for 5 min. The supernatant was filtered through a 0.20 µm syringe filter and transferred to autosampler vials. Samples were quantified using calibration standards prepared with MilliQ filtered water. The analysis was performed with a Waters UPLC coupled to a Sciex API5500 MS, operated under the following conditions:

**Table:** 

Ion source: electrospray positive	Column: UPLC HSS T3 2.1 × 100 mm, 1.8 µm
Injection volume: 10 µl	Flow rate: 0.45 ml/min
Mobile phases: A: 0.1% aqueous formic acid, B: acetonitrile	
Gradient: 0–4 min (100% A), 4–4.3 min (80% A), 4.3–5.5 min (0% A), 5.5–8 min (100% A)	

The transitions used for quantification were 90/62 and for confirmation 90/44. The transition for the internal standard was 95/63.

The “as received” WWB LOD was 20 ng/g. Concentrations of EC between the LOD and LOQ (60 ng/g) were estimated by Eurofins, using peak areas taken from the chromatogram but the uncertainty in these measurements was much greater than for concentrations > LOQ. This is due to the diverse matrix interference effects found across the range of market survey STPs. The same EC method was used for the experimental part of the investigation, but the LOD (10 ng/g) and LOQ (30 ng/g) were lower due to the use of the same basic, relatively simple product recipe used for all the test samples.

### Karl Fischer water

STP samples were analysed for their water content using Karl Fischer Coulometric analysis with a KEM MKC-500 analyser (Kyoto Electronics, Tokyo, Japan). Approximately 2 g of STP was accurately weighed into a 25 ml snap-top vial. 20.0 ml of methanol was added, and the sample sonicated for 15 min before being allowed to steep and settle for at least 2 h. A 100 μl aliquot of the methanol solution was injected into the Karl Fischer analysis cell. Water blanks were subtracted, and analyses conducted in triplicate.

### Nicotine, propylene glycol and glycerol

These compounds were determined by extracting 1.0 g of pre-moistened tobacco with 50 ml methanol (HPLC grade) containing heptadecane internal standard; the sample is shaken in a stoppered container for 3 h at 150 rpm. The extract is filtered through a 0.45 μm PVDF filter, and 1 μl of the filtered extract injected using a splitless injector. Separation occurred using helium carrier gas and a Phenomenex ZB-Waxplus (30 m × 0.53 mm i.d. × 1.00 μm) capillary column. The initial oven temperature was 120 °C, which was held for 4 min before temperature ramping at 20 °C/min to 230 °C with a 4 min final hold time; detection was by FID. Elution times were 7.01 min for *n*-heptadecane, 8.55 min for nicotine, and 11.01 min for glycerol.

### Nitrate nitrogen

Nitrate nitrogen was determined by aqueous extraction of 0.25 g tobacco in 25 ml deionised water with shaking at 180 rpm for 30 min. The extract is filtered through Whatman No. 40 filter paper prior to analysis using continuous flow analysis. Nitrate content of the STPs is analysed using reduction of the nitrate to nitrite with hydrazinium sulphate in the presence of copper (sulphate) catalyst, followed by reaction with sulphanilamide to form the diazo compound which is coupled with *N*-1-naphthylethylenediamine dihydrochloride to form a coloured complex, for which the absorbance is determined at 520 nm.

### Total nicotine alkaloids and total sugars

Total nicotine alkaloids and total sugars were analysed at BAT Southampton using continuous flow analysis. An aqueous extract of the ground STP (0.25 g in 25 ml deionised water) was prepared. The total sugars were calculated as the sum of reducing and non-reducing sugars, whereby reducing sugars were determined using methods described previously [6]. Non-reducing sugars were hydrolysed by the action of the enzyme invertase within the flow system, and the total non-reducing sugars then present were determined in a similar way. The total nicotine alkaloids were determined by reaction with sulphanilic acid and cyanogen chloride. The developed colour was measured at 460–480 nm.

### Water activity

2 g of each tobacco sample was placed into a disposable sample cup, which was inserted into a Labcell Ltd. Aqualab 3TE water activity meter. The measuring vessel is closed and readings taken. The Aqualab analyser was calibrated using saturated salt solutions (6 M NaCl and 0.5 M KCl).

### Sodium and chloride ions

Each STP sample was analysed for sodium and chloride in triplicate. One (± 0.1) g of STP was accurately weighed into a 50 ml labelled centrifuge tube. Forty (± 1) ml of fresh (equilibrated at room temperature) deionised water (18.2 MΩ) water was dispensed into each STP-containing centrifuge tube. The tubes were shaken for 1 h at 200 rpm on an orbital shaker and then centrifuged for 5 min at 4600 rpm. Each sample was diluted 100-fold by transferring 0.1 ml of centrifuged extract using a 100 μl Gilson pipette into a 40 ml plastic sterilin tube containing 9.9 ml of water and mixing thoroughly. The sample was transferred to a plastic 1.5 ml autosampler vial and capped. A sodium chloride stock solution was prepared by accurately weighing out between 33 and 36 mg of pure sodium chloride (> 99.9%, Fisher Certified Analytical Reagent, Fisher Chemicals, P/N: S/3160/53) directly into a 40 ml plastic sterilin pot. Deionised water (18.2 MΩ) was added using P10 and P5 ml air displacement Gilson pipettes, to give a 25 mM (1.461 mg/ml) solution. A 2.5 mM intermediate standard solution was prepared by diluting the stock solution by a factor of 10. The instrument was calibrated using working standard solutions of sodium chloride (with concentrations of 10, 25, 50, 100, 250 and 500 µM), prepared from the sodium chloride stock or intermediate working standards by appropriate dilution. The diluted extracts and calibration solutions were analysed with a Dionex ICS-3000 Ion Chromatography System. The reporting limit equates to 0.92 mg/g WWB for sodium ions and 1.42 mg/g WWB for chloride ions.

## Results

### Product survey

Results for EC concentrations in the STP samples are shown, product-by-product, in Additional file 1: Tables S1a and S1b, together with the other analytes measured in this study.

#### EC concentrations in commercial STPs

The concentrations of EC were below the LOD (20 ng/g WWB) for all the CT, DS, HP, SP, and plug products. In contrast, EC was detected in four of the ten L snus, 15 of the 22 P snus, and in 11 of the 16 MS products. Averages by category of STP product (on a WWB) were calculated by assigning values of LOD/2 (i.e. 10 ng/g) to samples that had levels of EC less than LOD [24]. EC averages and ranges of concentrations (in ng/g WWB) were as follows: P snus 28.1 (range < LOD–84); L snus 20.4 (range < LOD–37); MS 109 (range < LOD–688). When expressed on a DWB, concentrations in snus and MS approximately doubled in line with the moisture content of the STP. The results of the survey demonstrate that although EC was present in certain categories of STPs, the majority of samples in our study did not contain measurable concentrations.

#### Comparison with literature values

Literature reports of EC concentrations in tobacco, as outlined in the Introduction, are compared to those measured in the current study in Table 1. Our results, and those of Stepan et al. [22], both of which found no measurable EC in the majority of the analysed samples, demonstrate that EC is not ubiquitous in tobacco. The average WWB concentrations for EC in the MS samples we investigated are consistent with the concentrations found by Stepan et al. [22], and considerably lower (109 ng/g) than the 315 and 375 ng/g concentrations reported by Schmeltz et al. [14] for two Burley tobacco samples. However, it should be noted that there was a wide range of concentrations in our results for MS: from undetectable (< 20 ng/g) up to 688 ng/g. Thus, the tobacco samples for which EC has been reported in the literature are within the range found in our current study.

**Table 1:** Comparison of literature values for ethyl carbamate in tobacco to values measured in the current study

Tobacco type	Previous studies			The current study	
	Samples measured	[EC] (ng/g WWB)	References	Samples measured	[EC] (ng/g WWB)
Swedish snus	17 Lsnus	< 60 (DWB)	Stepan et al. [22]	10 Lsnus	< 20–37
	12 Psnus	< 60–284 (DWB)		22 Psnus	< 20–84
US moist snuff	15 MS	< 90	Oldham et al. [21]	16 MS	< 20–688
	CRP2	38			
Dry snuff	–	–	–	5 DS	< 20
Chewing tobacco	CRP 4	< 60 (DWB)	Stepan et al. [22]	13 CT	< 20
Hard pellet	–	–	–	2	< 20
Soft pellet	–	–	–	1	< 20
US snus	1	< 90	Oldham et al. [21]	–	–
Burley tobacco	2 Experimental samples	310, 375	Schmeltz et al. [14]	–	–
Cigarette blends	10 German blends	< 0.7	Schroth [18]	–	–
	3 German blends	1.4–2.9		–	–
	2 US blends	< 10	Clapp et al. [17]	–	–
	23 US blends	< LOD^a^	Teillet et al. [19]	–	–
Fine cut smoking tobacco (FCSA)	7 FCSA blends	< LOD^a^		–	–

^a^Unspecified

#### Variation within STP type and between manufacturers

Although EC was found in snus and MS products and not in the other styles of STP, differences between EC concentration were only significant (at 95% CI) between MS and CT. Further analysis showed that for snus there was no consistent significant difference (at 95% CI) in EC concentrations between manufacturers, which means that it is unlikely that a unique manufacturing step may be responsible for generating EC. For the MS samples, only the single PM brand, Marlboro Original, was significantly different from the other brands, and hence, for this sample, there may be a unique factor responsible for the high EC level measured.

#### Correlations between EC and other tobacco components

We measured a number of other components and properties of the STPs in this study: water content, water activity, nicotine, nicotine alkaloids, total sugars, propylene glycol, glycerol, and nitrate, sodium and chloride ions. These are shown in Additional file 1: Tables S1a and S1b. Concentrations of reducing sugars, ammonia nitrogen and pH have already been published for these STPs [6]. To identify factors that may be related to EC formation, the Pearson correlation coefficients (R) were calculated between the EC concentrations (WWB) and these parameters, all expressed on a WWB. These and the p values are shown in Table 2. The results in the first column were obtained by assigning a value of LOD/2 (i.e. 10 ng/g) to EC concentrations < LOD. Results in the second column included only brands for which EC > LOD.

**Table 2:** Correlations between ethyl carbamate and STP constituents

	Pearson correlation coefficient, R, and p value	
	All values included	Values < LOD excluded
All brands		
Karl Fisher water	0.285 (0.013)	0.223 (0.236)
All brands except US snus		
Karl Fisher water	0.274 (0.022)	0.223 (0.236)
Water activity	0.167 (0.167)	− 0.058 (0.762)
pH	0.125 (0.301)	− 0.222 (0.237)
Total nicotine alkaloids	0.087 (0.475)	0.270 (0.149)
Nicotine	0.131 (0.278)	0.219 (0.245)
Reducing sugars	− 0.167 (0.167)	− 0.188 (0.319)
Total sugars	− 0.176 (0.146)	− 0.189 (0.317)
Nitrate	0.029 (0.821)	0.641 (0.000)
Propylene glycol	− 0.169 (0.182)	− 0.621 (0.001)
Glycerol	− 0.341 (0.006)	− 0.329 (0.101)
Ammonia nitrogen	0.455 (0.000)	0.701 (0.000)
Chloride ion	0.368 (0.002)	0.348 (0.060)
Sodium ion	0.365 (0.002)	0.423 (0.020)

Correlations were calculated from wet weight basis concentrations

In the first column R was calculated by assigning a value of 10 ng/g to ethyl carbamate for values < LOD. In the second column R was calculated by excluding all values < LOD for ethyl carbamate

*LOD* limit of detection

**Fig. 2:**
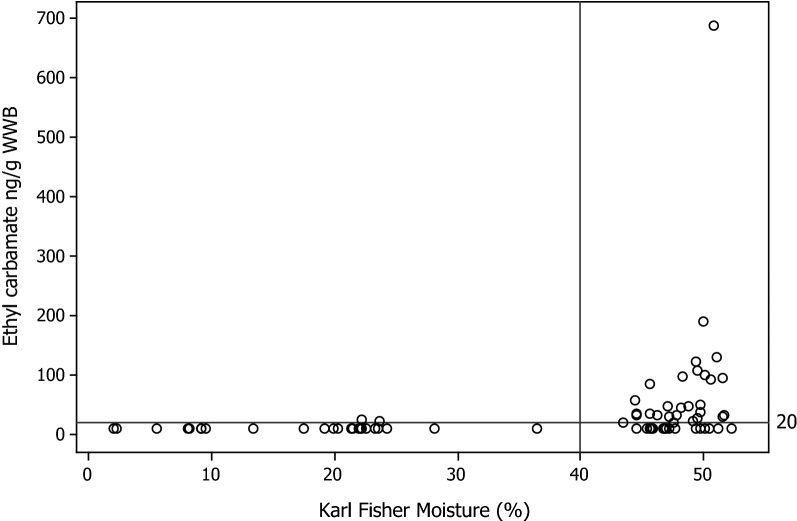
Ethyl carbamate (ng/g WWB) vs Karl Fisher water (%). The LOD is denoted by the reference line at 20 ng/g

**Fig. 3:**
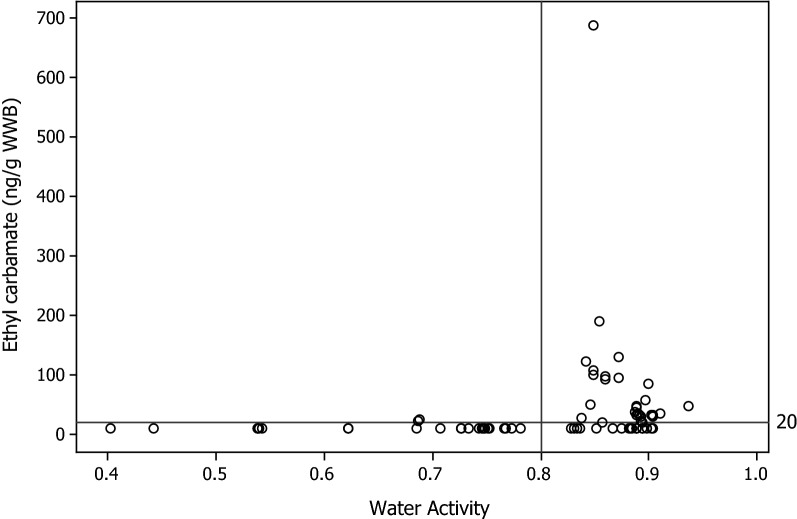
Ethyl carbamate (ng/g WWB) vs water activity. The LOD is denoted by the reference line at 20 ng/g

Across all the samples, there was a significant correlation (R = 0.285, p = 0.013) between Karl Fisher water content and EC concentration for all the brands in the study (Table 2). However, when only the values > LOD were tested the correlation failed to reach significance. This can be explained by examination of a plot of Karl Fisher water vs EC concentration (Fig. 2) which shows that almost all the STPs with measurable EC have water contents above 40%, but EC does not increase with increasing water content above this level. A similar pattern is observed for water activity (Aw), in which EC is only detected for brands with Aw > 0.8 (Fig. 3).

There were significant correlations between EC and glycerol (R = − 0.341), ammonia nitrogen (R = 0.455), chloride (R = 0.368) and sodium ions (R = 0.365) when EC concentrations < LOD were included. When samples with EC concentrations < LOD were excluded, water, glycerol, and chloride were not significantly correlated (p > 0.05) with EC. However, nitrate (R = 0.641), propylene glycol (R = − 0.621), ammonia nitrogen (R = 0.701) and sodium ions (R = 0.423) were significantly correlated.

### EC contents of experimental snus samples

Four specially manufactured snus products (snus A, B, C and D, as described in “Experimental” section) were used to test, in a controlled manner, the effects of a number of process and content parameters on EC concentrations. The aim of these experiments was to understand the relevance of processing, storage and chemical composition on EC concentrations in snus. Given that different STPs are processed in different ways and differ in their chemical compositions, findings of the snus study should not be extrapolated to other STP categories.

#### Processing and storage

##### The effect of processing conditions: pasteurisation, processing pH and moisture content

Baseline concentrations of EC were determined post-manufacture on tobacco samples A, B and C, which contained no added ethanol, urea or citrulline and were unaged (Additional file 1: Table S2). The samples ranged in moisture content from 33 to 55%, included both pasteurised and unpasteurised samples, and both with and without sodium carbonate. All samples had EC concentrations < LOD (i.e. < 10 ng/g).

##### Storage time

After storage for 4 and 12 weeks at 8 °C, all EC concentrations were also < LOD. The EC concentration of snus C was also < LOD after storage for 4 weeks at 20 °C (Additional file 1: Table S2). There was no difference between samples processed with moisture contents of 44 and 55%, no difference between samples processed with and without pasteurisation, and no influence of sodium carbonate. These results demonstrate no intrinsic EC formation by the standard snus product—consistent with the survey data on the F&L product.

##### Stability of EC in snus

To understand the stability of EC in snus, 200 ng/g of EC was added to samples of snus C and stored at 8 °C for 4 and 12 weeks, either in an open or in sealed glass containers. The snus EC concentrations after storage in the closed container (200.3 ng/g at 4 weeks and 193.3 ng/g at 12 weeks) were not significantly different (at 95%) to the level (200.0 ng/g) before storage, which suggests that EC is stable in the snus matrix. However, after storage of the snus in open containers there were significant reductions in the EC concentrations: 16% after 4 weeks and 71% after 12 weeks. These reductions were probably due to evaporative losses (Additional file 1: Table S3).

#### Impact of ingredients/constituents on EC concentrations in snus

##### Ethanol

One of the commonly cited pre-cursors of EC, ethanol, is generated in tobacco during curing, possibly by the actions of yeasts, and is also naturally present in cured tobacco leaf [25]. Although levels have not been quantified, naturally occurring ethanol could potentially react with other nitrogenous tobacco pre-cursors to form EC (Fig. 1).

Investigation of the role of ethanol in snus EC generation was conducted in two phases. In the first phase ethanol was added to portions of snus C in concentrations of 0.5, 1, 1.5, 2 and 4% and then stored for 4 weeks at 8 and 20 °C and 12 weeks at 8 °C. (Additional file 1: Table S4). Significant and linear increases in EC concentration were observed as ethanol concentrations increased. The increases were greater in the samples stored at 20 °C than in those stored at 8 °C. EC levels after 12 weeks at 8 °C were approximately double those found after 4-weeks storage.

**Fig. 4:**
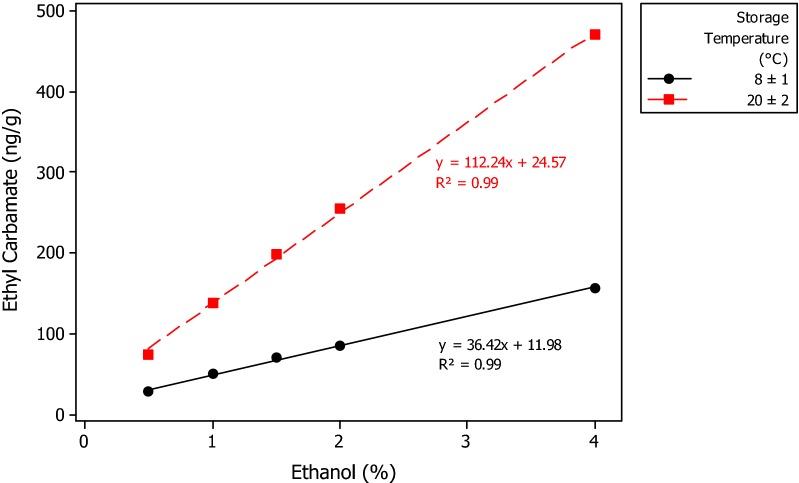
The effects of storage temperature and ethanol concentration on mean ethyl carbamate concentrations in an experimental STP after 24 weeks storage

**Fig. 5:**
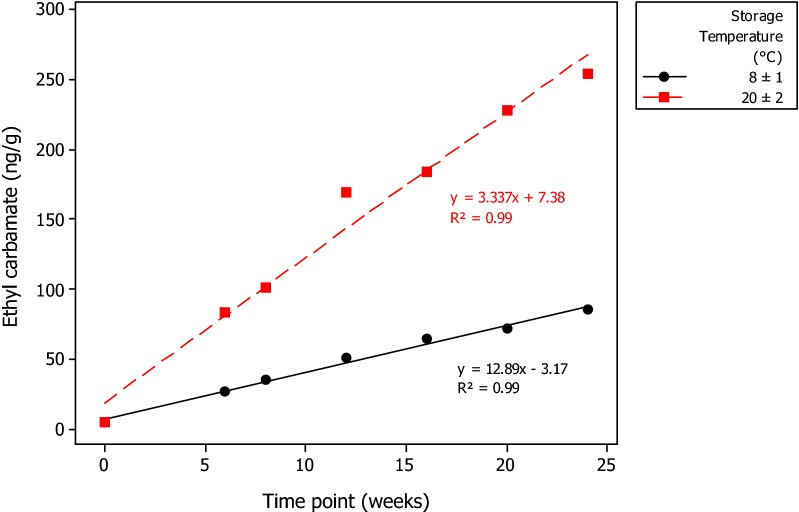
The effects of storage temperature and storage time on mean ethyl carbamate concentrations in an experimental STP containing 2% ethanol

Given the influence of ethanol on EC levels in these snus samples, a second phase experiment was conducted to better define the kinetics of EC generation. In the second phase experiment, snus samples with added ethanol were stored for up to 24 weeks at 8 °C or 20 °C (Additional file 1: Table S5). This longer-term study showed that EC continued to be formed over the 24-week storage period. EC concentrations after 24 weeks were linearly correlated with ethanol concentrations at both storage temperatures (for both, R^2^ = 0.99), as shown in Fig. 4. There were also linear correlations between storage times and EC concentrations. Figure 5 shows plots of EC concentration vs storage time for the samples containing 2% ethanol. Linear correlation coefficients were 0.99 and 0.98 for storage at 8 and 20 °C respectively. EC contents in samples stored at 20 °C were 3 ± 0.4 times higher than those stored at 8 °C.

##### Effects of urea and/or citrulline on EC concentrations

The two most commonly cited nitrogenous pre-cursors of EC in food-stuffs, urea and citrulline were also added at 1% to portions of snus C containing either 0 or 1% ethanol, and stored for 4 weeks at either 8 or 20 °C, and for 12 weeks at 8 °C before analysis for EC (Additional file 1: Table S6). The samples containing urea or citrulline without ethanol had EC concentrations < LOD, i.e. there was no effect on EC content. With 1% ethanol, the urea treated samples had mean EC concentrations not significantly different (at 95%) from those obtained by 1% ethanol treatment alone.

Similarly, the citrulline treated samples with 1% ethanol had mean EC concentrations not significantly different to those obtained by treatment with 1% ethanol alone (Additional file 1: Table S6). However, the mean EC concentration after storage at 20 °C (32.7 ng/g) was 18% lower than obtained by treatment with only ethanol (39.7 ng/g). This difference was significant at 95%. The EC concentration in the sample with 1% ethanol and 1% citrulline stored for 12 weeks at 8 °C (17.7 ng/g) was significantly lower (at 95%) than that in the 1% ethanol sample with no added citrulline (20.3 ng/g).

Urea and citrulline were also added together at 1% to samples of snus C containing 4% ethanol (Additional file 1: Table S7). One of the snus samples had a moisture of 55%, while the other had been dried to 15% prior addition of these compounds. The EC concentrations were measured after 4 weeks at 20 °C and compared with EC concentrations in a sample with only 4% ethanol and no urea or citrulline. The EC concentrations in the 55% moisture content samples treated with urea and citrulline were significantly (at 95%) lower than the 4% ethanol comparator. EC levels in the 15% samples were not significantly different.

These results show no positive contribution of citrulline or urea to EC formation in STPs and suggest a possible countering effect with citrulline.

##### Snus water content

For snus containing 4% ethanol (but no other additives) and stored for 4 weeks at 20 °C there was no significant difference in EC concentrations in the product containing 55% moisture compared with the same product dried to 15% before storage (Additional file 1: Table S7). Similarly, for snus containing 4% ethanol and 1% urea and 1% citrulline there was no significant difference (at 95%) in EC concentrations after storage at 20 °C between the product at 55% moisture and that at 15% moisture.

##### Snus pH

Snus D treated with citric acid to obtain a pH of 5.5 but with no ethanol, urea or citrulline had an EC concentration < LOD, as did the pH 8.5 comparator. When treated with 4% ethanol, snus D at pH 5.5 had an EC concentration of 28 ng/g, which was significantly lower than in a comparable sample of snus D at pH 8.5 (114 ng/g—Additional file 1: Table S8).

## Discussion

### Mechanisms for EC formation in tobacco

The observed variation in levels of EC, both between and within different styles of STP is intriguing. In this section we discuss possible mechanisms for EC formation in light of both the product survey results and those of the controlled snus experiments.

#### STP processing

##### Fermentation

Fermentation is an established environment in which EC can be generated in food and alcoholic beverages. The role proposed by Schmeltz et al. [14] for fermentation in the generation of EC in tobacco and smoke echoes the mechanisms used to explain formation of EC in foodstuffs. Two of the STP styles investigated in the current work, DS and MS, undergo fermentation steps as part of their manufacture (Table 3). During tobacco fermentation, the tobacco is moistened and microbes and/or enzymatic activity modifies its chemical composition.

**Table 3:** Characteristics of different types of STP

	Primary tobacco types used	Fermented	Pasteurised	Sodium chloride*	Sodium or potassium carbonate (%)	Pack water* (%)	Humectant*	Sugar*	pH*
MS	Dark fire-cured and air cured burley	Yes	No	Yes	< 1%	ca 50	0–4.36%	No	6.4–8.4
DS	Dark fire cured and air cured burley	Yes	No	Small amount	ca 2%	< 10	0–0.24%	No	8.1–9.5
Swedish snus	Air-cured burley and sun-cured Oriental	No	Yes	Yes	ca 2%	ca 50	PG (L and P: 2–3.5%), glycerol (L only: 1–3%)	No	7.5–9.4
CT	Air cured cigar tobacco, burley and/or dark fire cured	No	No	Small amount	No	ca 20	Glycerol (ca 3%)	23–40%	5.6–6.5
Plug	Air-cured burley and/or dark fire cured	No	No	N/D	No	18	Glycerol (1.7%)	15%	5.3
HP	N/D	No	No	No	N/D	2	No	5%	8
SP	N/D	No	No	No	N/D	13	No	5%	5.3

Levels are reported on a wet weight basis

*N/D* not determined or unknown, *MS* moist snuff, *DS* dry snuff, *CT* chewing tobacco, *HP* hard pellet, *SP* soft pellet, *L snus* loose snus, *P snus* portion snus, *PG* propylene glycol

* Data are from this study. If not maked information taken from Klus et al. [49] and Wahlberg and Ringberger [50].

However, the results of this work and that of Stepan et al. [22] do not support fermentation as an important source of EC in STPs. EC was not detected in any sample from one fermented product style (DS) in either study, whereas it was detected in some samples of MS in both studies. If fermentation was a critical mechanism, it could be expected that EC would be seen in all fermented samples, unless there are significant differences in fermentation steps between these product categories or processes used by manufacturers. Additional file 1: Table S9 shows the blend composition of the STP CRPs, but offers little obvious alternative explanation for the substantial differences in EC contents between DS and MS. Furthermore, our study demonstrated measurable EC levels in a significant number of Swedish snus products—which do not undergo fermentation during their production. We therefore conclude that fermentation is not a critical step for EC formation in STPs.

##### Pasteurisation

Temperature is also a factor leading to the presence of EC in food. Studies of EC formation in bread and puddings [12], in wine [26, 27] and in soy sauce [13] have shown that concentrations increase rapidly with temperature. It is therefore plausible that the pasteurisation process conducted during snus manufacture, which involves holding tobacco at high temperatures, contributes to EC formation from pre-established precursors within the tobacco. However, the experiments on experimental snus samples conducted in this work showed no impact of pasteurisation on EC levels. Moreover, while there were measurable concentrations in some of the commercial Swedish snus samples, other Swedish snus samples showed no EC content. Clearly, were pasteurisation an important parameter it would be expected that EC would be seen in most if not all snus samples. Finally, EC was also seen in MS samples where high temperature pasteurisation does not take place. We therefore conclude from these observations that the elevated temperature conditions used in manufacture of some STPs is not in itself a critical step in EC formation.

##### Snus processing moisture and pH

Our measurements with experimental snus samples showed no sensitivity to tobacco pH or moisture content during processing. However, these observations are limited to snus, and cannot be extrapolated to other STPs.

##### EC stability in storage

Finally, our experiments have shown that EC, although chemically stable in snus, is sufficiently volatile that significant amounts can evaporate from open containers over a period of several weeks.

### Chemical composition of STPs

#### Ethanol

As discussed above, ethanol, is generated during curing, and is present in cured tobacco leaf [25]. It is therefore a plausible precursor for EC as shown in Fig. 1.

In the experimental study on snus, the only samples in which there were detectable concentrations of EC were those that contained added ethanol. The effect of added ethanol on EC concentrations was striking. Even with the lowest concentration of ethanol (0.5%) used in the study a significant concentration of EC (27 ng/g) was generated in the snus after 24 weeks at 8 °C. However, the molar conversion of ethanol to EC observed in these experiments was low, at 10^−3^–10^−4^ %. There were also clear, linear, temperature- and time-dependent increases in EC concentrations as ethanol concentrations increased from 0.5 to 4%. For example, for the 24-week period, raising the storage temperature from 8 to 20 °C increased EC concentrations in all ethanol-containing snus samples threefold. This implies an activation energy of the order of 63 kJ/mol.

As discussed above, the findings from the snus experimental study cannot be extrapolated to other STP categories, due to differences in their processing and composition. However, to understand the possible relevance of the findings from our laboratory snus studies to the wider range of commercial STPs, we examined available composition data on STP manufacturers’ websites. Our search confirmed that ethanol is added to some STPs as an ingredient, or as a processing aid. For example, the ingredient data sheets provided by the US Tobacco (UST) arm of Altria [28] shows that for UST products ethanol is an ingredient in MS, but not in DS manufactured by UST. Swedish Match provides percentage compositions of their Swedish snus products [29]. Ethanol is not amongst the quantified ingredients, but it is disclosed as a processing aid in their STPs. EC was quantified in 11 of the 14 Swedish Match P snus products, but in only two of the six L snus products analysed in this study. Finally, the Fiedler and Lundgren products measured in this study were ethanol-free [30], and EC was not detected in these products.

Therefore, this limited inspection of commercial STP composition suggests that ethanol addition may be an important factor leading to EC generation in those STPs it is found in. The concept that the addition of a known EC-precursor to an STP during manufacture would result in increased levels of EC in STPs is logical in principle and would point to the predominant formation-stage of EC as post-manufacture, during the product shelf-life. If, as seems likely, there is significant EC production in STPs post-manufacture, then the age of the sample at the time of analysis will be a contributing factor to the levels of EC measured in these samples, as found previously with the acrylamide contents of STPs [5]. As the age of the STP at the time of analysis is an uncontrollable variable in the type of product survey conducted in this study, it would be manifest as unexplained variation in the measurement data—consistent with the observations of this study.

We also assessed potential errors in our product survey measurements arising from EC generation in storage post-sampling and pre-analysis. Use of the activation energy estimate of 63 kJ/mol, and an EC production rate of 3.5 ng/g/week for a 2% addition of ethanol predicts a low level of EC 0.2 ng/g/week at the − 20 °C storage temperatures used. Over the approximately 3-month period between sampling and analysis, we would expect 2–3 ng/g EC to develop, which is small in comparison to the values measured for STPs containing EC.

#### Nitrogenous species

Some of the nitrogenous precursors involved in the formation of EC in foods and alcoholic beverages are also present in cured tobacco. During curing, tobacco proteins break down to amino acids and other soluble nitrogen compounds. In particular, relatively high concentrations of the acid amide, arginine, are formed during air curing of tobacco, [31] probably by the action of tobacco enzymes on glutamine or proline. As curing progresses and the leaf structure is compromised, microbes enter the leaf structure and arginine is hydrolysed with the loss of ammonia to form citrulline. Urea, which can be formed by the catabolism of arginine, has also been reported in Burley tobacco [32].

##### Citrulline and urea

Addition of two different nitrogenous precursors, urea and/or citrulline, failed to generate detectable levels of EC in snus even after storage under the same conditions. The addition of urea and/or citrulline to the ethanol containing snus did not increase levels of EC. In fact, there were some indications that addition of citrulline may decrease EC concentrations. Clearly, there are sufficient levels of nitrogenous precursors in the tobacco that the ethanol concentration is the rate-limiting factor in the formation of EC. The identity of these nitrogenous precursors is unclear, however the product survey provided some insights as to the relative importance of various nitrogenous constituents of tobacco. The lack of impact from urea or citrulline addition suggests that either there are considerably more reactive precursors present in tobacco, or substantially greater quantities than the 1% levels of urea/citrulline added in this study; of these two possibilities the first appears more likely.

##### Other nitrogenous components of tobacco

One of the major nitrogenous compounds in tobacco is nicotine. However, the product survey showed no correlation of EC concentrations with nicotine, or total nicotine alkaloids. In contrast, the survey showed significant correlations between EC and ammonia nitrogen (R = 0.455) across all STPs (the correlation increases (R = 0.701) when only brands with measurable levels of EC are considered), and nitrate when products < LOD were excluded from the analysis. The first correlation is consistent with the generation of ammonia during the enzymatic and microbial changes to tobacco during curing and possibly fermentation, particularly formation of arginine. This may point to an important role of tobacco processing on the generation of EC nitrogenous precursors, rather than EC itself. An alternative nitrogenous precursor was proposed by Schmeltz et al. [14], who originally hypothesized that EC in tobacco leaf and smoke may be formed from maleic hydrazide used as a plant growth regulator on tobacco. However, tobacco treated with maleic hydrazide did not contain more EC than untreated tobacco. The authors therefore concluded that EC formation in tobacco was unrelated to maleic hydrazide.

#### Storage water content

A notable observation within this study was that the styles of STP with measurable EC (P snus, L snus and MS) had, on average, higher moistures (42–49%) than those that did not (HP—2%, DS—9%, SP—13%, Plug—17 and CT—22%). EC was therefore only observed in this study in products with a water content > 22%. Our data also showed a similar effect with water activity, where those products with measurable EC levels all had water activities > 0.8 (Fig. 3). However, it should be noted that some products with Aw > 0.8, and water content > 22% had no detectable levels of EC. These observations led to a significant but weak correlation (R = 0.285, p = 0.013) between EC and moisture content across all survey STPs (Table 2). However, EC content was not correlated with water content or Aw amongst only those STPs containing EC.

As reactions between ethanol and nitrogenous EC precursors are aqueous reactions, the level of free water within the tobacco/STP matrix could dictate the hydrolytic solvation properties within the STP, and therefore potentially the rate of solution-phase reactions. Above threshold levels, where sufficient free water is available to allow solvated reactions to occur, changes in water level would be unimportant. This hypothesis supports some but not all of the observed trends in EC content between STPs of differing water content, and also differences in EC content between DS (and Swedish snus) and MS. However, inconsistent with the solvation mechanism hypothesis, in the experiments with experimental snus samples reducing moisture from 55 to 15% had no effect on generation of EC during storage of snus containing 4% ethanol over a period of 4 weeks. Critically, the 15% water content experimental snus samples containing EC were drier than those commercial samples, that did not contain EC.

#### pH

Although there was no significant correlation between pH and EC concentrations from the survey results, pH differed between those categories of commercial STP that showed no detectable EC levels (CT and DS—which are the most acidic at pH 6.1), and those that did (snus and MS—which have a more alkaline pH, averaging 8.5 and 7.8 respectively). Within STP category there was no trend between STP pH and EC content. The experimental snus samples showed a dramatic effect of tobacco pH; lowering the pH from 8.5 to 5.5 reduced EC concentrations fourfold in ethanol-containing snus. This suggests that pH is a critical parameter in EC generation when ethanol is present, based upon the experimental snus samples. As an understanding of this observation, it is plausible that more acidic pH’s may retard EC formation by protonating and ‘protecting’ the amine groups of nitrogenous tobacco precursor(s). Protonation of amines occurs at tobacco pHs with nicotine being a well-studied example [33].

#### Other STP components

Another major difference between styles with and without EC is the salt level. As shown in Table 3, Swedish snus and MS have higher salt loadings than other styles of STP. This is reflected in significant (p < 0.05) correlations between EC and sodium (R = 0.365) and chloride (R = 0.368) ions. High salt levels are also present in soy sauce, which is notable for the presence of significant concentrations of EC [9]. However, it is not clear if, and how, sodium and chloride ions may be involved in EC formation, other than indirectly as a marker for higher moisture. Glycerol is significantly and negatively correlated (R = − 0.341) with EC across all samples of STPs. It is not used in P snus, DS or MS (except for 2 brands). However, it is added to L snus brands (Table 3) and many of these have measurable amounts of EC. Glycerol, being hygroscopic can act to lower Aw, alternatively, these observations may be simple association between the presence of EC in some STPs and common ingredients, rather than mechanistically relevant factors.

### Conclusions as to the mechanism for EC generation in STPs

Interpretation of our survey findings has suggested a mechanism for the presence of EC in STPs is base-mediated conversion of ethanol via nitrogenous compounds in tobacco. EC content of experimental snus samples increased with time after application of ethanol and was noticeably temperature dependent. The nitrogenous precursors in tobacco have not been identified, but often-cited food precursors to EC, urea and citrulline, were not important reactants in our study. Previously proposed processing factors, including fermentation and high temperature tobacco processing such as pasteurisation, showed no impact on EC levels, although they may possibly influence the generation of nitrogenous precursors in tobacco. This mechanism is consistent with the observations of the current, and previous studies. However, while the observations by Schmeltz et al. [14] of EC in Burley tobacco, and by Oldham et al. [21] in a reference MS product, may reflect this mechanism, for example via ethanol content arising during leaf processing, they may also point to additional relevant factors not identified in the present study.

### Exposure to EC from STP use

Like foods and beverages, exposure of consumers to EC from STP use will depend on its concentration in the STP and the level of STP consumption by the consumer. However, for STPs there are two other factors to consider that are not usually relevant for foods and beverages. Firstly, since the STP is not itself ingested, we have to determine the amount of EC extracted from the STP during use. Secondly, with specific reference to snuffs and chewing tobaccos, the amount of expectoration that occurs with use must also be assessed. These factors are considered in the following paragraphs in order to estimate exposure of STP users to EC.

#### Daily consumption

Several studies have reported Swedish snus consumption amongst a population of STP users. Andersson et al. [34] found the average daily consumption of Swedish portion snus was 14.4 g snus/day among 23 users of portion snus, and 20.8 g snus/day among 22 users of loose snus. In a much larger study [35], 2914 snus users reported average daily consumptions of 11–12 g/day for portion snus and 29–32 g/day for loose snus.

Maxwell [36] estimated average MS consumption amongst US users in 1980 as 7.3 g/day (one and one-half 34 g tins per week). The Surgeon General’s 1986 report on smokeless tobacco assumed a rate for MS of 10 g/day [37]. In 1988, Hatsukami et al. [38] reported an average consumption of 12.4 g/day amongst male adult consumers of US MS. Hecht et al. [39, 40] reported an average consumption of 20.4 g/day (4.2 tins per week) of MS (mainly Copenhagen, Skoal and Kodiak brands). Hecht et al. [41] also reported a considerably lower consumption of 5.3 g/day (1.1 ± 0.8 tins/week). The average of these daily consumption values is 11.1 g/day.

#### Extraction

The amount of an STP constituent extracted during use is termed mouth level exposure or MLE, which is often reported as the percentage of the constituent extracted during use. MLEs have not been reported in the literature for EC. However, a range of values for other water-soluble constituents has been published. Digard et al. [42] determined MLEs for a range of Swedish snus constituents. The most water-soluble such as nicotine, propylene glycol and TSNAs, chloride, sodium, ammonium and nitrate ions, had mean extractabilities ranging from 24 to 38% after 1 h of use. Caraway and Chen [43] obtained similar results for users of a US snus. They found average levels of nicotine extraction of 39%, and average TSNA extraction levels in the range 9.5–30% depending on the particular TSNA. With extraction of soluble constituents from snus not exceeding 40%, we would expect EC, which is also water-soluble, to have similar extractability. Unfortunately, no data are available for the extraction of constituents from other STPs during use.

#### Expectoration

Snus in Sweden is routinely placed in the upper lip and consumers do not expectorate, but users of snuff and chewing tobacco in the US generally expectorate during use, which would tend to reduce exposure to extracted STP contaminants such as EC. To our knowledge, the only study of toxicant losses due to expectoration was a study of NNK exposure in 15 MS users [41]. The NNK in the expectorated saliva as a proportion of the initial amount in the MS portion ranged from 0 to 48.7% with an average of 14.2%.

#### Exposure

We have estimated average exposures to EC from use of Swedish snus using the concentrations found in the present study, together with the average consumption from Digard et al. [35], and an estimated extraction efficiency for EC of 40% based on published data for other water-soluble STP components. These are tabulated in Table 4.

**Table 4:** Estimated exposures (µg/person/day) to EC from Swedish snus and American MS

STP	Mean EC by STP style (ng/g)	Consumption (g/day)	Estimated average extraction of EC (%)	Estimated expectoration losses (%)	Estimated EC Exposure (µg/day)
Swedish P snus	28.1	11.5	40	0	0.13
Swedish L snus	20.4	30.7	40	0	0.25
US MS	109	11.1	40	14	0.41

Estimated exposures to EC amongst Swedish portion snus consumers are, on average, 0.13 µg/day, whereas Swedish loose snus consumers would be exposed to an average of 0.25 µg/day. For MS, exposure was estimated using the average of reported consumption rates (11.1 g/day) and using a value of 14% for losses through expectoration [41]. This gives an average estimate for exposure to EC from MS as 0.41 µg/day. Users of CT, DS and pellet products will be exposed to levels lower than these estimates for Swedish snus and US MS.

These amounts would be in addition to the amounts of EC obtained from dietary sources, which are discussed in the next section.

#### Comparison to exposure from other sources

As mentioned in the Introduction the main contributors to dietary EC (excluding alcoholic beverages) are fermented products such as soy sauce, bread (especially when toasted), yogurts and cheeses. The Joint FAO/WHO Expert Committee on Food Additives (JECFA) has estimated that food products in general (excluding alcoholic beverages), contribute on average less than 1 µg EC per person per day [10]. Therefore, on average, consumers of STPs appear to be exposed to EC levels (≤ 0.41 µg/day) lower than reported average dietary exposure (1 µg/day). In addition the European Food Safety Authority (EFSA) has estimated the contribution of alcoholic beverages to EC exposure, which can be substantially higher than from STP use. Based on survey data from various European countries and based on median EC levels found in European beverages, drinkers at the 95th percentile level of consumption who drank exclusively beer (1000 ml/person/day), wine (417 ml/person/day) or spirits (125 ml/person/day) increased EC exposure by 0–5, 2.1 and 2.6 μg/person/day, respectively. For consumers of stone fruit brandy at the 95th percentile level (125 ml/person/day), EC exposure increased by 32.5 μg/person/day.

### Risk characterisation

In 2005 a conference of the European Food Safety Authority (EFSA) evaluated several approaches for estimating health risks from contaminants that are both genotoxic and carcinogenic [44, 45]. The margin of exposure (MOE) was the preferred approach but it was emphasized that it could be used to prioritise risk management actions but could not be used to evaluate health risk itself. The MOE is a ratio between a benchmark dose (a reference point derived from either experimental or epidemiological dose–response data, usually selected as a 10% response) and the specific human exposure. With higher values of MOE representing lower risk, MOEs greater than or equal to 10,000 are generally considered a low priority for risk management actions [44, 46, 47].

EFSA has specifically used the MOE approach, with a benchmark dose (BDML) of 0.3 mg/kg BW/day, to determine the level of concern that should be accorded to the presence of EC in foods and alcoholic beverages [10]. Use of EFSA MOE figures allows for the calculation that exposures to EC totalling less than 1.8 µg per person per day would correspond to an MOE of 10,000 or more, and hence would not be a high priority for risk management. It was estimated that a maximum dietary exposure excluding alcoholic beverages was 1 μg EC/person per day (equivalent to an MOE of 18,000) which is therefore well below the threshold for concern. Assessing the impact of average exposure to EC amongst STP users from Swedish snus or US MS, in addition to food exposure, shows that total daily exposure remains substantially below the threshold exposure level of 1.8 µg per person per day. Similarly, exposure to EC through use of the other STPs examined in this study will not substantially increase exposure to EC beyond food-based exposure. According to the standard approach with MOE calculations, EC content of STP should therefore be regarded as a low priority for risk management actions [44, 46, 47].

## Conclusions

Our survey of Swedish and US STPs found that the majority (60%) examined, including all the CT, DS, plug and pellet products, did not have detectable EC levels (i.e. < 20 ng/g WWB). Only three of the seven categories of STP (MS, L snus and P snus) contained detectable levels of EC. Within these three categories, a significant percentage of products had EC concentrations < LOD (41% of the snus products and 31% of the MS products). Using estimated EC concentrations (LOD/2) for products with EC < LOD gave mean concentrations for these three categories of 109, 20 and 28 ng/g WWB for MS, L snus and P snus respectively. However, the difference in average EC concentrations between the snus and MS styles of STP was not statistically significant. Levels of EC across all the STPs examined in this study were significantly and positively correlated with levels of moisture, ammonia nitrogen, sodium and chloride and negatively correlated with glycerol. The presence of EC was limited to STPs with moistures greater than 40% and Aw greater than 0.8, and to styles of STP with higher pH.

Controlled laboratory experiments using experimental snus samples provided valuable insights into factors leading to EC formation. The experiments showed unequivocally that, within the experimental parameters, none of the ethanol-free snus samples had detectable levels of EC and that addition of ethanol was necessary for the formation of EC. We also found that addition of nitrogenous precursors that have been associated with EC formation in other products did not increase EC concentrations in snus. The effect of ethanol on EC formation was enhanced by increases in storage time and temperature, was faster at higher pH conditions, but was not affected by moisture content. The role of fermentation and high temperature processing such as pasteurisation did not appear to be important in the production of EC. Nitrogenous pre-cursors to EC appear to be naturally present in tobacco, but their identity remains unclear.

Using published consumption rates for STPs and mouth level exposures to STP components we estimate that consumers of MS, DS, CT, pellet products and Swedish snus with average levels of EC would be exposed to levels lower than those present in the normal diet. MOE calculations suggest that these levels would not be considered a health concern to the consumer. Even without factoring in the proportion extracted during use, Rodu and Jansson [2] showed that exposures to lead, cadmium, polonium, formaldehyde and benzo(a)pyrene from use of STPs were consistent with normal dietary exposure, and concluded that these contaminants were not a health concern to STP users. We can now add EC to this list.

## Additional file

**Additional file 1. ** Additional Tables.

## Abbreviations

CT: chewing tobacco
DWB: dry weight basis
DS: US dry snuff
EC: ethyl carbamate
FDA: US Food and Drug Administration
HP: hard pellet
LOQ: limit of quantification
LOD: limit of detection
L snus: Swedish loose snus
MOE: margin of exposure
MS: US moist snuff
P snus: Swedish portion snus
SP: soft pellet
STP: smokeless tobacco product
UPLC/MS/MS: ultra performance liquid chromatography tandem mass spectrometry
WWB: wet weight basis
